# Neomycin: sources of contact and sensitization evaluation in 1162 patients treated at a tertiary service^⋆^

**DOI:** 10.1016/j.abd.2022.07.008

**Published:** 2023-03-30

**Authors:** Maria Antonieta Rios Scherrer, Érica Possa Abreu, Vanessa Barreto Rocha

**Affiliations:** a Hospital das Clínicas, Universidade Federal de Minas Gerais, Belo Horizonte, MG, Brazil; b Department of Internal Medicine, Faculty of Medicine, Universidade Federal de Minas Gerais, Belo Horizonte, MG, Brazil

**Keywords:** Allergic contact dermatitis, Drug allergy, Neomycin

## Abstract

**Background:**

Neomycin is used in several over-the-counter pharmaceutical formulations in Brazil. In Europe and Canada, where it is not freely available, its sensitization frequency is lower than in the United States, where this does not occur.

**Objective:**

To present the frequency of sensitization to neomycin observed in a tertiary hospital and the pharmaceutical formulations sold in Brazil containing neomycin.

**Method:**

Retrospective analysis of positive results to neomycin, obtained through patch tests performed in a tertiary hospital, from 2009 to 2018 and investigation of topical drugs and vaccines containing neomycin in Brazilian databases available on the internet.

**Results:**

Among 1,162 patients, 71 (6%) had positive reactions to neomycin, 65% female and 35% male individuals, 46% were over 50 years old, and 24% had a personal history of atopy. The dermatitis lasted from four months to 20 years. Lesions were located in 69% of the patients on the upper limbs, in 55% they were on the lower limbs, and in 42% they were disseminated in more than 4 sites. Polysensitization was detected in 55% of cases. Of these, 28% were linked to sensitization to rubber allergens and 27% to potassium bichromate. A total of 158 topical presentations of neomycin were found: 79 ointments, 58 creams, 10 ophthalmic solutions, seven otological solutions, one oral solution, two nasal solutions, and one antiseptic powder, in addition to 11 types of vaccines.

**Study limitations:**

Retrospective study.

**Conclusion:**

Sensitization to neomycin occurred in 6% of the studied population, affecting more females aged over 50 years, with skin lesions located mainly on the upper and lower limbs, in the context of chronic contact dermatitis. Neomycin was found in 135 formulations, most of them available over the counter, as well as in 11 miscellaneous vaccines.

## Introduction

Neomycin is an antibiotic that belongs to the aminoglycoside family and blocks bacterial protein synthesis through the irreversible binding to the 30S ribosomal nuclear subunit. It is effective against anaerobic bacteria, staphylococci, and Gram-negative bacteria, except *Pseudomonas aeruginosa.* After prolonged use, bacterial resistance may develop. Its percutaneous absorption is minimal, and it is used in topical formulations for skin, ear, and eye treatment, in solutions for urinary instillation, solutions for peritoneal irrigation, dental and veterinary treatment, and in animal feed and fish tanks. Through the oral route, it is used to sterilize the intestine before surgery and to treat hepatic coma. Traces of this substance are present in several vaccines.^1^, ^2^

In Brazil and the United States, it can be purchased without a prescription, which causes a high frequency of sensitization, unlike in European countries, where this frequency is low.^1^, ^3^, ^4^

## Methods

A total of 1,162 patients with suspected contact dermatitis underwent patch tests between October 2009 and October 2018. The Brazilian Standard Battery was used, consisting of 30 substances (FDA-allergenic, RJ, Brazil) using Finn chambers®. (Oy, Finland) applied to the back of patients with readings at 48 and 96 hours. Reactions were graded as +, ++ and +++ following the International Contact Dermatitis Research Group (ICDRG) criteria.

The data were collected in an Excel® table, with the selection of those positive for neomycin, which were analyzed regarding demographic aspects, location of lesions, time of evolution, personal history of atopy, association with other allergens and relevance based on the clinical history and exposure to the agent. An internet search was also carried out on generic and similar brand drugs, in addition to vaccines containing neomycin available in our environment. These drugs were categorized according to the active principles associated with neomycin and then redistributed according to the vehicles available on the market.

## Results

Among 1162 patients, 71 (6%) had positive reactions to neomycin, 46 (65%) were female and 25 (35%) male individuals, 21 (29.5%) were Caucasians and 50 (70.5%) non-Caucasians. Age ranged from 15 to 77 years (mean 53.5), with 33 (46%) being older than 50 years (Table 1). Seventeen (24%) had a personal history of atopy. The dermatitis lasted from four months to 20 years, with 77.5% of the cases being lasting up to five years. Forty-nine (69%) had lesions on the upper limbs, 39 (55%) on the lower limbs, followed by the cervical region and dorsum of the hands, which were both affected in 28 (39%). Twelve patients (17%) had lesions on the eyelids, eight (11%) on the ears and none on the anogenital area. Thirty (42%) were disseminated, with the involvement of four or more sites (Table 2). Thirty-two (45%) were positive from one to three allergens, and 39 (55%) were positive to more than three. Current relevance was established in ten patients (14%) and considered as past relevance in 20 (28%). Twenty cases (28%) had allergic contact dermatitis to rubber as their main diagnosis, with 17 (24%) having a rubber-related occupational nature. Nineteen patients (27%) were sensitized to potassium bichromate, of which 11 (15%) were occupational, related to cement (Table 3).

**Table 1:** Demographic data of 71 patients sensitized to neomycin.

	n (%)
**Female sex**	46 (65)
**Male sex**	25 (35)
**Caucasians**	21 (29.5)
**Non-causasians**	50 (70.5)
**Atopy**	17 (24)
**Age: 17 to 77 years**	33 (46)
**> 50 years**

**Table 2:** Most frequent sites of ACD to neomycin.

Site	Number	Percentage
Upper limbs	49	69
Lower limbs	39	55
Cervical region	28	39
Hands	28	39
Eyelids	12	17
Ears	8	11
Anogenital region	0	0
Disseminated (> 4 sites)	30	42
**Total**	166

**Table 3:** Polysensitization.

Allergens	Number	Percentage
**Rubber allergens**	**20**	**28**
Occupational nature	17	24
**Potassium bichromate**	**39**	**27**
Occupational nature	11	15
**Total**	59	55

Regarding the substances, neomycin was present not only in isolated formulations but also associated with other active principles, which are also frequent allergens, such as corticosteroids, other antibacterial agents, antifungals, anesthetics and steroids, as shown in Table 4. As for the commercial products, Table 5 provides a list of those that contain neomycin, in addition to the composition of each item. Fifty-eight creams, 79 ointments, ten ophthalmic solutions, seven otological solutions, one oral solution, two nasal solutions, and one antiseptic powder were found.^5^, ^6^, ^7^

**Table 4:** Topical medications containing neomycin.

Category	Composition	Total
Total	Neomycin sulfate	1
Antifungal	Neomycin sulfate + Thiabendazole	1
Antibiotic	Neomycin sulfate + Zinc Bacitracin	2
	Neomycin sulfate + Zinc Bacitracin + Zinc oxide + Zinc peroxide + Polymyxin B Sulfate	
Steroid	Neomycin sulfate + Clostebol acetate	1
Anesthetic	Neomycin sulfate + Sodium bismuth tartrate + Procaine hydrochloride	3
	Neomycin sulfate + Bismuth sodium tartrate + Procaine hydrochloride + Menthol	
	Neomycin sulfate + Lidocaine hydrochloride + Hyaluronidase	
Corticoid	Neomycin sulfate + Dexamethasone disodium phosphate	5
	Neomycin sulfate + Betamethasone valerate	
	Neomycin sulfate + Fludroxycortide	
	Neomycin sulfate + Deoxymethasone	
	Neomycin sulfate + Dexamethasone acetate	
Corticoid + antibiotic	Neomycin sulfate + Prednisolone Acetate + Polymyxin B Sulfate	3
	Neomycin sulfate + Hydrocortisone + Polymyxin B Sulfate	
	Neomycin sulfate + Dexametasona + Polymyxin B Sulfate	
Antibiotic + antifungal + antiparasitic	Neomycin sulfate + Polymyxin B Sulfate + Nystatin + Tinidazole	1
Vasoconstrictor + corticoid	Neomycin sulfate + Naphazoline Hydrochloride + Dexamethasone Disodium Phosphate	1
Corticoid + antibiotic + antifungal	Neomycin sulfate + Triamcinolone Acetonide + Gramicidin + Nystatin	4
	Neomycin sulfate + Ketoconazole + Betamethasone dipropionate	
	Neomycin sulfate + Encetoconazol + Betamethasone dipropionate	
	Neomycin sulfate + Nystatin + Dexametasona + Tyrothricin phosphate	

Sources: consultas.anvisa.gov.br, 2022; Topical Antimicrobials. Medscape, 2022.

**Table 5:** Topical presentations with neomycin.

Vehicle	Composition	Total
Cream	Neomycin sulfate + Clostebol acetate	
	Neomycin sulfate + Ketoconazole + Betamethasone dipropionate	
	Neomycin sulfate + Betamethasone valerate	
	Neomycin sulfate + Deoxymethasone	
	Neomycin sulfate + Triamcinolone Acetonide + Gramicidin + Nystatin	
	Neomycin sulfate + Ketoconazole + Betamethasone dipropionate	
	Neomycin sulfate + Encetoconazol + Betamethasone dipropionate	
	Neomycin sulfate + Nystatin + Dexamethasone + Tyrothricin phosphate	
	Neomycin sulfate + Polymyxin B Sulfate + Nystatin + Tinidazole	
		9
Ointment	Neomycin sulfate	
	Neomycin sulfate + Zinc Bacitracin	
	Neomycin sulfate + Thiabendazole	
	Neomycin sulfate + Hydrocortisone Acetate + Troxerutin + Ascorbic Acid + Benzocaine	
	Neomycin sulfate + Ketoconazole + Dipropionato de betametasona	
	Neomycin sulfate + Betamethasone valerate	
	Neomycin sulfate + Deoxymethasone	
	Neomycin sulfate + Fludroxycortide	
	Neomycin sulfate + Dexametasona + Polymyxin B Sulfate	
	Neomycin sulfate + Triamcinolone acetonide + Gramicidin + Nystatin	
	Neomycin sulfate + Ketoconazole + Betamethasone dipropionate	
	Neomycin sulfate + Encetoconazol + Betamethasone dipropionate	
		12
Ophthalmic suspension	Neomycin sulfate + bismuth sodium tartrate + procaine hydrochloride + Menthol	
	Neomycin sulfate + Prednisolone acetate + Polymyxin B sulfate	
	Neomycin sulfate + Hydrocortisone + Polymyxin B sulfate	
	Neomycin sulfate + Dexametasona + Polymyxin B sulfate	
		4
Otological solution	Neomycin sulfate + Lidocaine hydrochloride + Hyaluronidase	
	Neomycin sulfate + Sodium bismuth tartrate + procaine hydrochloride	
	Neomycin sulfate + fluocinolone acetonide + Polymyxin B sulfate + procaine hydrochloride	
	Neomycin sulfate + Encetoconazol + Betamethasone dipropionate	
	Neomycin sulfate + Dexamethasone disodium phosphate	
		5
Oral solution	Neomycin sulfate + Sodium bismuth tartrate + procaine hydrochloride + menthol	
	Neomycin sulfate + Sodium bismuth tartrate + procaine hydrochloride	
		2
Nasal solution	Neomycin sulfate + Naphazoline Hydrochloride + Dexamethasone disodium phosphate	
		1
Antiseptic powder	Neomycin sulfate + Zinc Bacitracin + Zinc oxide + Zinc peroxide + Polymyxin B Sulfate	
		1

Sources: consultas.anvisa.gov.br, 2022; Topical Antimicrobials. Medscape, 2022.

As for the vaccines, Table 6 shows the 11 immunizers that contain neomycin in their composition, either in the form of traces, as an excipient, among the residues, or among the other constituents.^8^, ^9^, ^10^

**Table 6:** Vaccines containing neomycin on the composition.

Name	Composition
Influenza vaccine (fractionated, inactivated)	Inactivated, fragmented and purified *Myxovirus influenzae* virus strains grown in embryonated chicken eggs. Traces of neomycin or polymyxin, gentamicin and thimerosal as preservatives.
Varicella vaccine	Live attenuated virus from the Oka strain. Each dose of the vaccine must contain at least 1,350 Plaque Forming Units (PFU) of attenuated varicella zoster virus (VZV). May contain gelatin and traces of neomycin, kanamycin and erythromycin.
Measles, mumps, rubella vaccine (Triple viral vaccine)	Live (attenuated) viruses from Wistar RA 27/3 strains of rubella virus, Schwarz measles, and RIT 4385, derived from Jeryl Lynn mumps. Excipients: human albumin, lactose, sorbitol, mannitol, neomycin sulfate and amino acids.
aDTP-IPV/Hib	Components of the triple acellular bacterial vaccine (aDTP), contains a component of the bacterium *Haemophilus influenzae* type b conjugate vaccine and inactivated (dead) poliovirus types 1, 2 and 3. The composition also includes: lactose, sodium chloride, 2-phenoxyethanol, hydroxide of aluminum and water for injection. Traces of antibiotics (streptomycin, neomycin and polymyxin B), formaldehyde and bovine serum albumin.
aDTP-IPV-HB/Hib vaccine	Acellular triple bacterial vaccine (aDTP) components, *Haemophilus influenzae* type b conjugate bacterial component, inactivated (dead) poliovirus types 1, 2, and 3, and hepatitis B virus surface component.
	Components: lactose, sodium chloride, 2-phenoxyethanol, aluminum hydroxide and water for injection. Traces of antibiotics (streptomycin, neomycin and polymyxin B), formaldehyde and bovine serum albumin.
Hepatitis A adsorbed vaccine (inactivated)	Each dose of the vaccine contains 720 U. EL./0.5 mL or 1,440 U. EL./1.0 mL of hepatitis a virus (HAV) antigens.
	Excipients: aluminum hydroxide, polysorbate 20, amino acids, disodium phosphate, monopotassium phosphate, sodium chloride, potassium chloride and water for injections.
	Residue: neomycin sulfate.
Rabies vaccine for human use	Inactivated rabies virus, maltose (stabilizer), human albumin.
	Diluent: Sodium chloride solution. Traces of streptomycin, neomycin and/or polymyxin B.
Oral Polio Vaccine (OPV)	Bivalent attenuated oral vaccine consisting of poliovirus types 1 and 3. It also contains magnesium chloride, sucrose, neomycin, streptomycin or erythromycin (stabilizers) and red amaranth or phenol purple (pH indicator stain).
Inactivated Polio Vaccine (IPV)	Trivalent injectable vaccine, consisting of particles of poliovirus types 1, 2 and 3. It contains 2-phenoxyethanol, polysorbate 80, formaldehyde, Hanks 199 medium, hydrochloric acid or sodium hydroxide. It may contain traces of neomycin, streptomycin and polymyxin B, used during production
Hepatitis A and B combined vaccine	Inactivated (dead) hepatitis A and hepatitis B virus surface protein. Other ingredients: aluminum salts, formaldehyde, neomycin sulfate, phenoxyethanol, polysorbate 20, sodium chloride, water for injection.
Herpes zoster vaccine	Live attenuated virus vaccine. Each 0.65 mL dose contains 19,400 PFU (Plaque Forming Units) of Varicella Zoster Virus (VZV) of the Oka/Merck strain. Sucrose, gelatin (hydrolyzed porcine) and traces of neomycin.

Sources: Anvisa, 2022; Brazilian Ministry of Health, 2014; SBBIM, Instituto Butantan, 2019.

## Discussion

The prevalence of positive neomycin patch tests in the general population is around 1%. Neomycin sensitization is shown to be decreasing in Europe, ranging from 1.1% to 3.8%, with a mean of 2.6%.^1^, ^2^ It had previously reached rates of 10.2% in Croatia and 18.4% in children from Portugal. In the United States, however, sensitization varied from 7% to 13% in the last two decades, with an average of 11.4%.^3^, ^11^ Canada has also shown a downward trend in this rate, influenced by the reduction in its availability to the public, sold only by medical prescription.^12^ In Brazil, data from 2009 showed a frequency of 5.7%, very similar to what was found between 2009 and 2018 (6%), but during this period there were no changes in the rules for obtaining this medication as an over-the-counter drug.^13^ It should be noted that the present data were collected with final readings at 96 hours, despite the known possibility that late reactions to neomycin can occur up to the seventh day.

Overall, ointments are less sensitizing than creams and lotions, because they contain fewer preservatives, emulsifiers, and solvents. However, studies show that the sensitizing properties of neomycin are, to some extent, determined by the vehicle. Surfactants in creams and lotions could reduce drug sensitization and, on the contrary, ointments, as they promote occlusion, would increase its absorption and consequently sensitization. Therefore, these authors recommend the use of neomycin in creams, lotions, or powders to reduce sensitization risk.^2^, ^11^

A study carried out in western Canada on the prevalence of neomycin sensitization in this region showed a predominance in females of 82.1%, whereas the data in the present study showed a prevalence of 65%, confirming the trend of greater sensitization in females. The mean age of patients was 49.5 years, with most being aged between 40 and 50 years, not much different from the present study.^1^, ^2^ According to other studies, allergic contact dermatitis to topical medications usually becomes more common with advancing age.^3^

The evolution of eczema was chronic in all cases, ranging from four months to five years in 77.5%. A study with data related to sensitization to topical drugs in Brazil, including neomycin, showed similar results.^13^

Publications on allergic contact dermatitis to topical medications show the involvement of the legs mainly, highlighting the presence of stasis dermatitis and leg ulcers. The present data showed that the upper limbs were the most affected, followed by the lower limbs, which could be explained by the association with occupational allergic contact dermatitis to rubber and cement. Other mentioned risk sites related to contact dermatitis to topical drugs are the eyelids and perianal region. The authors detected 17% of lesions on the eyelids, 11% on the ears, and no involvement of the anogenital area, although formulations for these regions were found in the authors’ country. It should be noted that 42% of the patients in the present study had disseminated body lesions.^11^, ^12^, ^13^

The factors predisposing to allergy to topical medications, including neomycin, include certain locations, such as the lower extremities, skinfolds, ears, use of occlusive dressings, transdermal medications, and pre-existing dermatoses. Among these are chronic venous insufficiency, external otitis, post-surgical wounds, chronic eczematous lesions, as observed here associated with allergic contact dermatitis to rubber and cement.^3^, ^4^, ^12^, ^13^, ^14^, ^15^

Occupational dermatitis to neomycin usually affects the hands and face and occurs in healthcare professionals, pharmacists, dentists and veterinarians, which was not observed in the present series.^1^

Polysensitization, defined as the presence of positive reactions to three or more unrelated haptens, was mainly described in studies carried out with topical medications in general, mainly in patients older than 70 years.^14^, ^15^, ^16^ In the present study, positivity to more than three allergens was found in 55% of the cases.

Neomycin consists of four sugar rings linked by glycosidic bonds, and one of these rings is common to almost all aminoglycosides. Therefore, it shows cross-reactions with most of them, such as amikacin, gentamicin, kanamycin, tobramycin, paromomycin, and butyrosine. Streptomycin and spectinomycin have a peculiar structure, different from the others, occurring at a lower rate. Thus, the percentage of cross-reactions to neomycin is 90% for paromomycin and butyrosine (not available in Brazil), 70% for framycetin, 60% for tobramycin and kanamycin, 50% for gentamicin, and 4% for streptomycin.^17^

As neomycin is often associated with bacitracin in the same topical preparations, sensitization or co-reaction to both is not uncommon, suggesting that both antibiotics could act synergistically to elicit an immune response. Formulations with three antibiotics associating polymyxin B with neomycin and bacitracin are described. As polymyxin B is not part of the standard patch test batteries, its frequency of sensitization is unknown.^1^, ^17^ When investigating the identified formulations, the authors detected two associations with bacitracin in ointment and antiseptic powder vehicles and seven with polymyxin B. The latter is associated with corticoids and antifungals in several presentations, including creams, ointments, otological solutions, ophthalmic suspensions, and antiseptic powders.

Small amounts of neomycin absorbed from the gastrointestinal tract can trigger the dissemination of dermatitis, the onset of lesions in previous dermatitis sites, reactivation of the positive reaction to the patch test, or disseminated contact dermatitis. The administration of other aminoglycosides can also cause this condition in patients sensitized by topical exposure to neomycin. Vaccination with preparations containing neomycin could theoretically cause the appearance of systemic reactions. However, as the elicitation threshold for reactions is 100 to 1000 µg of neomycin, and the concentration found in vaccines is 25 µg, it is expected that it may cause a minimal, transient, and localized reaction at the injection site, and therefore is not a contraindication to vaccination.^1^ A history of anaphylactic reactions to neomycin would be a contraindication to its use.

The limitation of this study refers to the fact that it is a retrospective study.

## Conclusion

In this study, sensitization to neomycin was present in 6% of the studied population, affecting more frequently women aged over 50 years, mainly on the upper and lower limbs, in the context of chronic contact dermatitis. Neomycin was found in 135 formulations, most of them available without a prescription, in addition to 11 different vaccines.

Reduction in the free availability of these drugs, together with careful prescriptions, will lead to a decrease in the sensitization rate to the European levels, with neomycin use being reserved for strictly indicated cases.

## Financial support

None declared.

## Authors’ contributions

Vanessa Barreto Rocha: Design and planning of the study; critical review of important intellectual content; collection, analysis and interpretation of data; approval of the final version of the manuscript, effective participation in research orientation.

Érica Possa Abreu: Design and planning of the study; data collection, or data analysis and interpretation; drafting of the manuscript; collection, analysis and interpretation of data; critical review of the literature; approval of the final version of the manuscript.

Maria Antonieta Rios Scherrer: Design and planning of the study; data collection, or data analysis and interpretation; statistical analysis; drafting of the manuscript or critical review of important intellectual content; collection, analysis and interpretation of data; effective participation in research orientation; intellectual participation in the propaedeutic and/or therapeutic conduct of the studied cases; critical review of the literature; approval of the final version of the manuscript.

## Conflicts of interest

None declared.
